# Supplementary material for: Ultrafast power doppler ultrasound enables longitudinal tracking of vascular changes that correlate with immune response after radiotherapy
Source: Theranostics. 2024 Oct 21;14(18):6883–96. doi: 10.7150/thno.97759 (PMC11610147; doi:10.7150/thno.97759)
Supplement: Supplementary file 1 — Supplementary figures and table. [file thnov14p6883s1.pdf]

Ultrafast power doppler ultrasound enables longitudinal tracking of vascular changes that correlate with immune response after radiotherapy

Shannon E. Martello[1], Jixin Xia[2], Jiro Kusunose[3], Benjamin C. Hacker[1], McKenzie A. Mayeaux[2], Erica J. Lin[4], Adrienne Hawkes[3], Aparna Singh[3], Charles F. Caskey[3], Marjan Rafat[1,2,5]*

[1]Department of Chemical and Biomolecular Engineering, Vanderbilt University, Nashville, TN, USA

[2] Department of Biomedical Engineering, Vanderbilt University, Nashville, TN, USA

[3] Department of Radiology, Vanderbilt University Medical Center, Nashville, TN, USA

[4] Department of Biological Sciences, Vanderbilt University, Nashville, TN, USA

[5]Department of Radiation Oncology, Vanderbilt University Medical Center, Nashville, TN, USA

*Author for correspondence: Marjan Rafat, PhD, Engineering and Science Building, Rm. 426, Vanderbilt University, Nashville, TN 37212. Phone: (615) 343-3389, Fax: (615) 343-7951, E-mail: marjan.rafat@vanderbilt.edu

Supplemental Figures

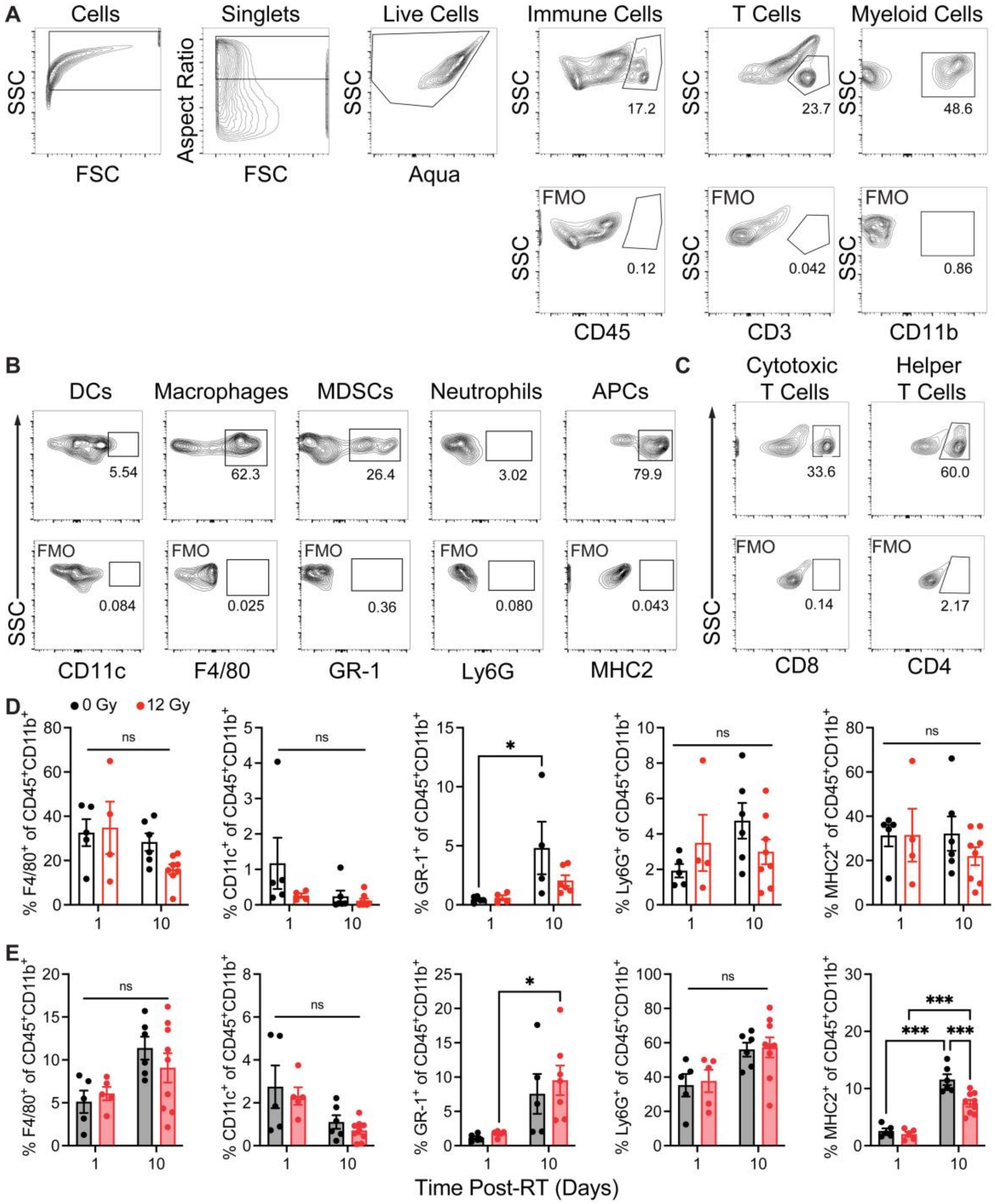

Figure S1. Tumor-infiltrating immune cell flow cytometry analysis. A. Full gating strategy for myeloid and T cells in 67NR and 4T1 tumors with full minus one (FMO) controls shown in the bottom panels. **B.** Representative gating for myeloid subpopulations. **C.** Representative gating for T cell subpopulations. Percentage of myeloid subpopulations, including F4/80^{+} macrophages, CD11c^{+} dendritic cells, GR-1^{+} myeloid-derived suppressor cells, Ly6G^{+} neutrophils, and MHC2^{+} antigen presenting cells, in **(D)** 67NR tumors (0 Gy: n=5 for 1 day, n=4 for 10 day; 12 Gy: n=4 for 1 day, n=6 for 10 day) and **(E)** 4T1 tumors (0 Gy: n=5 for 1 day, n=6 for 10 day; 12 Gy: n=5 for 1 day, n=7 for 10 day) 1 and 10 days after 12 Gy RT exposure. Error bars represent standard deviation with *p<0.05 and ***p<0.001 as determined by ANOVA.

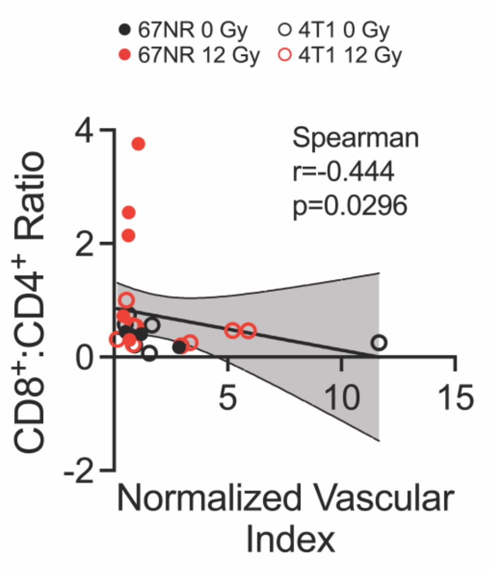

Figure S2. CD8⁺:CD4⁺ T cell ratio 10 days post-RT inversely trends with vascular index 1 day after RT. Spearman correlation and linear regression analyses of the 10 day post-RT tumor $CD8^+$:$CD4^+$ T cell ratio and 1 day post-RT vascular index (normalized to day -1). Each point represents one mouse with n=4 mice for 67NR 0 Gy, n=5 mice for 67NR 12 Gy, n=6 mice for 4T1 0 Gy, and n=9 mice for 4T1 12 Gy.

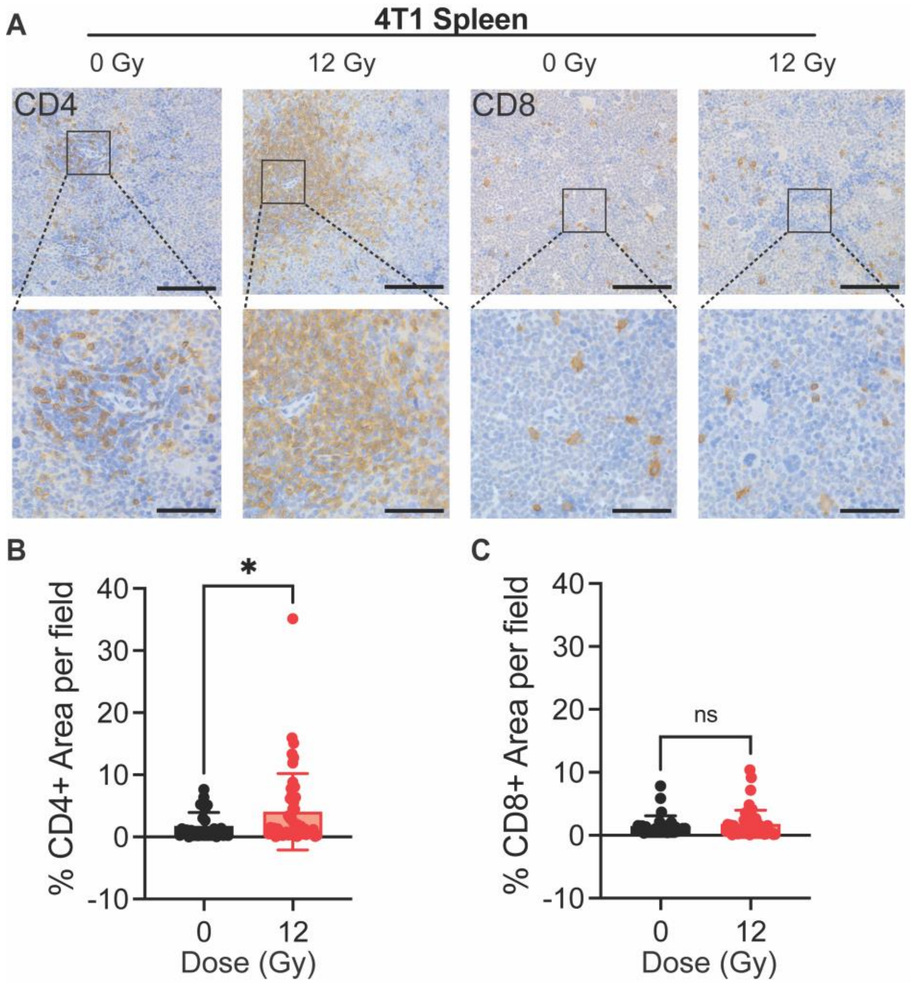

Figure S3. Immunohistochemistry (IHC) validates T cell population shifts in spleens from mice with 4T1 tumors post-RT. A. Representative images of $CD4^+$ and $CD8^+$ IHC staining of spleens in mice with 4T1 tumors at 10 days post-RT. Scale bars are 100µm (top row) and 50 µm (bottom row). Quantification of the percent area of **(B)** $CD4^+$ and **(C)** $CD8^+$ staining in spleens at 10 days post-RT. n=3 mice per condition. Each point represents one field of view with 10 total fields per mouse. Error bars indicate standard deviation with *p<0.05 determined by unpaired t-test.

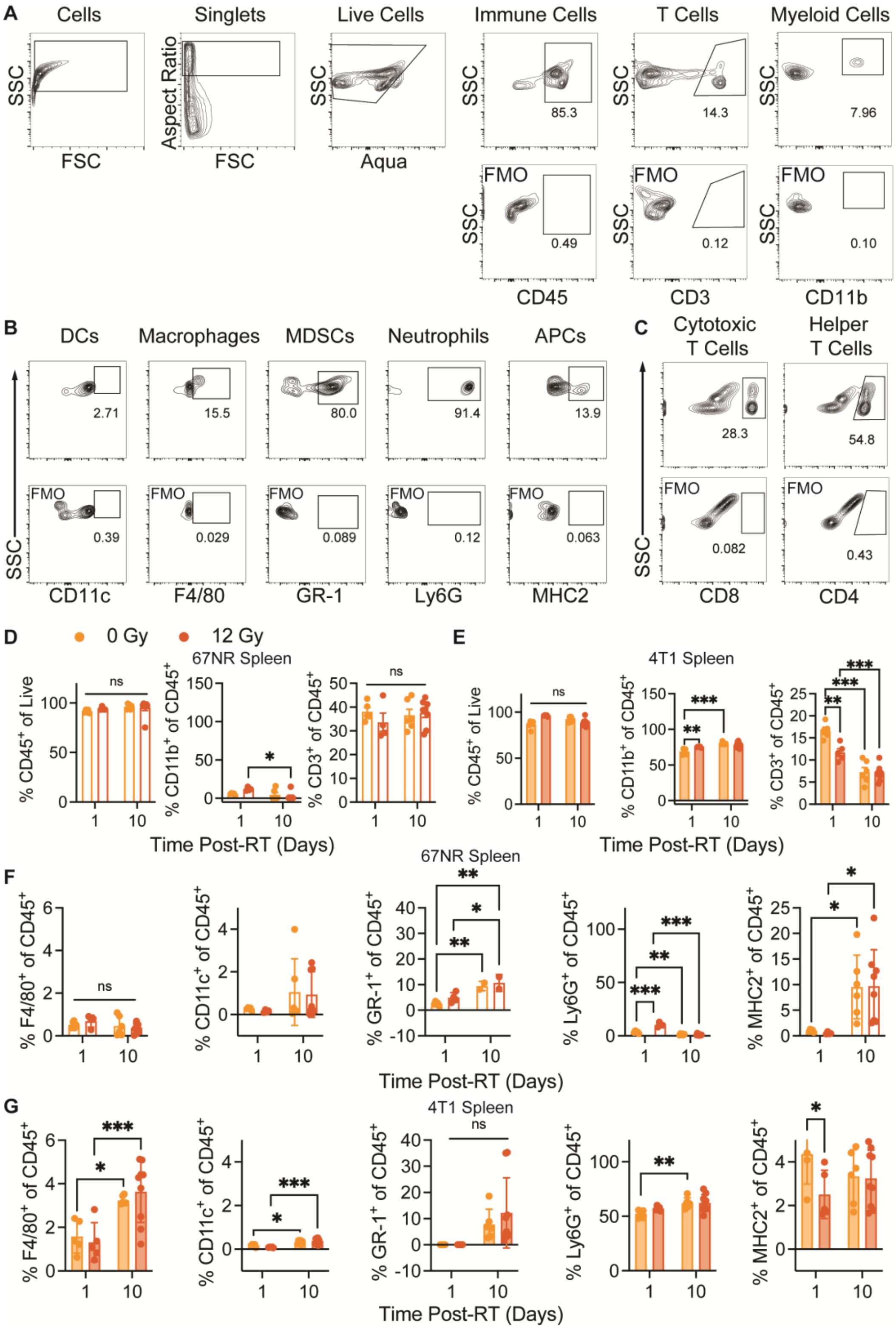

Figure S4. Flow cytometry analysis of splenic immune cell populations. A. Full gating strategy for myeloid and T cells in spleens with full minus one (FMO) controls shown in the bottom panels. **B.** Representative gating for myeloid subpopulations. **C.** Representative gating for T cell subpopulations. Percentage of $CD45^{+}$ immune, $CD11b^{+}$ myeloid, and $CD3^{+}$ T cell populations in spleens from **(D)** 67NR and **(E)** 4T1-bearing mice 1 and 10 days after 12 Gy RT exposure of the

tumor. Percentage of myeloid subpopulations in the spleens of **(F)** 67NR and **(G)** 4T1-bearing mice, including F4/80$^+$ macrophages, CD11c$^+$ dendritic cells, GR-1$^+$ myeloid-derived suppressor cells, Ly6G$^+$ neutrophils, and MHC2$^+$ antigen presenting cells. For 67NR, 0 Gy: n=5 mice for 1 day, n=4 for 10 day; 12 Gy: n=4 for 1 day, n=6 for 10 day. For 4T1, 0 Gy: n=5 mice for 1 day, n=6 for 10 day; 12 Gy: n=5 for 1 day, n=7 for 10 day. Error bars represent standard deviation with *p<0.05, **p<0.01, and ***p<0.001 as determined by ANOVA.

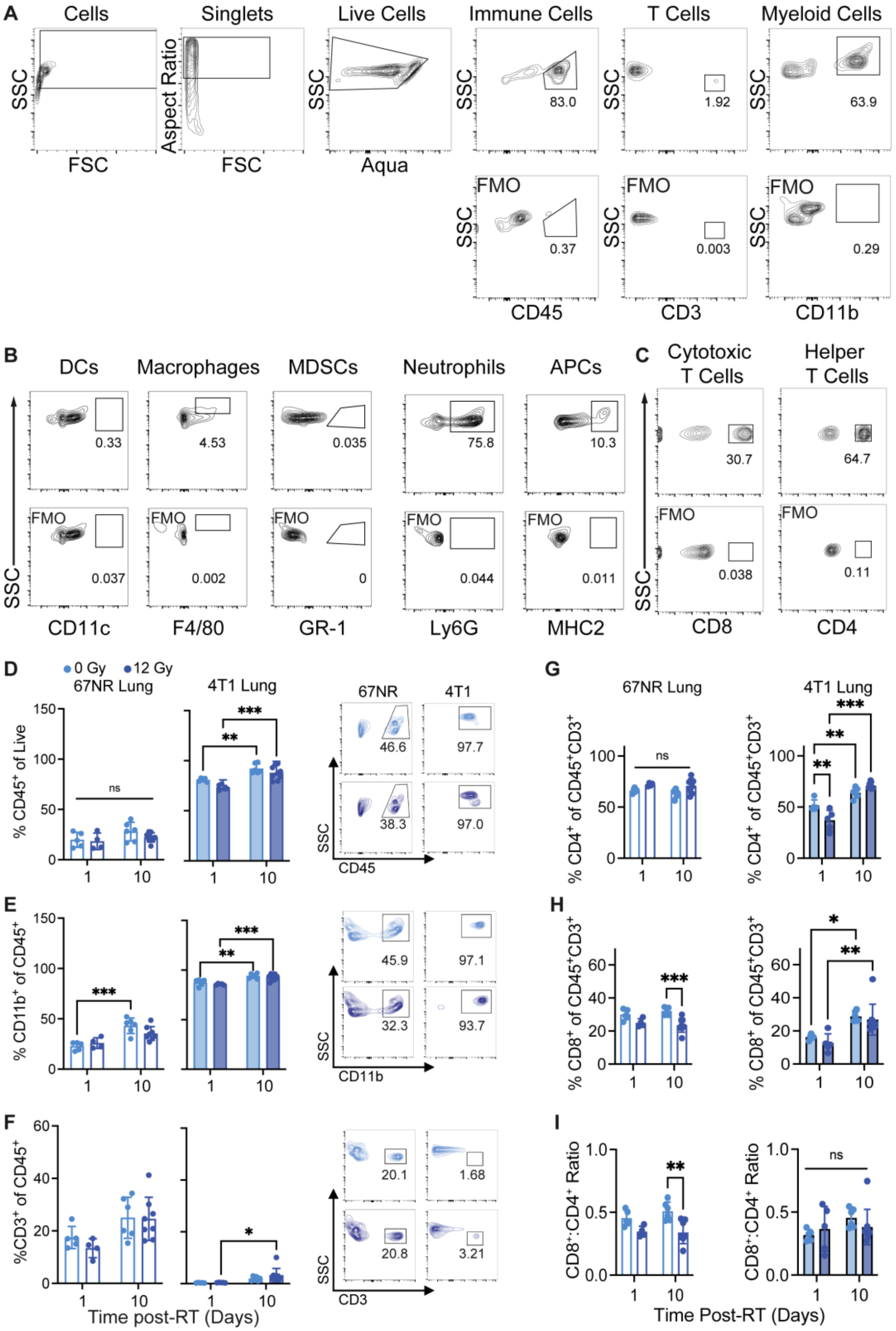

Figure S5. Flow cytometry analysis of lung immune cell populations. A. Full gating strategy for myeloid and T cells in lungs with FMO controls shown in the bottom panels. **B.** Gating strategy for myeloid subpopulations. **C.** Gating strategy for T cell subpopulations. Flow cytometry characterization and representative contour plots of lung immune cell populations at 1 and 10 days post-RT, including **(D)** CD45^{+} immune cells, **(E)** CD11b^{+} myeloid cells, and **(F)** CD3^{+} T cells

in lungs of mice with 67NR (left; 0Gy: n=5 for 1 day, n=4 for 10 day; 12 Gy: n=4 for 1 day, n=6 for 10 day) and 4T1 (right; 0 Gy: n=5 for 1 day, n=6 for 10 day; 12 Gy: n=5 for 1 day, n=7 for 10 day) tumors. Error bars represent standard deviation with *p<0.05, **p<0.01, and ***p<0.001 as determined by two-way ANOVA. Percentage of **(G)** $CD4^+$ helper T cells, **(H)** percentage of $CD8^+$ cytotoxic T cells, and **(I)** ratio of $CD8^+$:$CD4^+$ T cells calculated from absolute counts of $CD45^+CD3^+CD4^+$ and $CD45^+CD3^+CD8^+$ cells in the lungs of mice bearing 67NR and 4T1 tumors lungs 1 and 10 days after 12 Gy RT exposure of the tumor. For 67NR, 0 Gy: n=5 mice for 1 day, n=4 for 10 day; 12 Gy: n=4 for 1 day, n=6 for 10 day. For 4T1, 0 Gy: n=5 mice for 1 day, n=6 for 10 day; 12 Gy: n=5 for 1 day, n=7 for 10 day. Error bars represent standard deviation with *p<0.05, **p<0.01, and ***p<0.001 as determined by ANOVA.

Table S1. Linear Regression Analysis[a].

Condition	Slope		Intercept		n
	m	95% CI	m	95% CI	
67NR 0 Gy	0.739	0.528 to 0.950	17.11	-41.46 to 75.68	22
67NR 12 Gy	0.237	0.139 to 0.334	92.84	68.23 to 117.4	20
4T1 0 Gy	0.865	0.608 to 1.122	-28.94	-95.31 to 37.43	32
4T1 12 Gy	0.716	0.501 to 0.932	-4.959	-66.44 to 56.53	39

[a]m=mean; CI=confidence interval; n=number of measurements with 9 mice included in each of the 67NR 0 and 12 Gy groups, 15 in 4T1 0 Gy, and 13 in 4T1 12 Gy
